# Greater frequency of premature birth when both parents do not acknowledge filiation

**DOI:** 10.1186/s13052-014-0071-9

**Published:** 2014-08-13

**Authors:** Renato Lucchini, Mario De Curtis, Francesco Franco, Domenico Di Lallo

**Affiliations:** 1 Department of Paediatrics and Neuropsychiatry, University of Rome La Sapienza, Rome, Italy; 2 Regional Health Agency of Lazio, Rome, Italy

## Abstract

No abstract

## 

The recent economic crisis that has been affecting Italy in the last few years has led to an increase in poverty and to worse social conditions, inevitably affecting infancy ([1], [2]). The health conditions of babies, notably worse among those belonging to needy families, can be influenced by their status already before birth. Babies born to women living in disadvantaged conditions, such as immigrant women, and who have therefore little access to national health services during pregnancy, are exposed to greater disease risk ([3]). Another risk condition for the newborn is the failure to acknowledge parenthood by both parents. In the Lazio region in the last eight years, as many as 436,255 babies were born; those whose filiation was not acknowledged or was acknowledged by the mother only were 9401 (2.2%). Compared to newborns whose filiation was acknowledged by both parents, these children presented a higher rate of premature birth (<37 weeks)-11.9 vs. 7.9%- p < 0.001; very low birth weight (<1500 g)-2.1 vs. 0.9%- p < 0.001 (Figure 1) and late first prenatal visit (>12 weeks) - 11.4 vs. 2.1% p < 0.001. We can hypothesize that the increased risk observed in newborns whose filiations was not acknowledged by either parents, as those born to immigrants, depends on a cluster of conditions associated to the mothers’ social economic and cultural disadvantaged conditions during pregnancy (lack of regular working conditions, heavier workloads, inappropriate diet and hygienic conditions, poor housing, inadequate or delayed obstetrical care). All women and their children should be guaranteed equal access to health services during pregnancy and delivery, regardless of ethnicity and social status, with equal dignity and guarantee of safety. To this regard, the Italian law warrants full right to health care during pregnancy and delivery, although there is need to improve information on the services dispensed to women during pregnancy, also with the aim to overcome the feelings of distrustfulness that induces many women to avoid referral to obstetrical care during pregnancy and thus have an unhealthful lifestyle.

**Figure 1:**
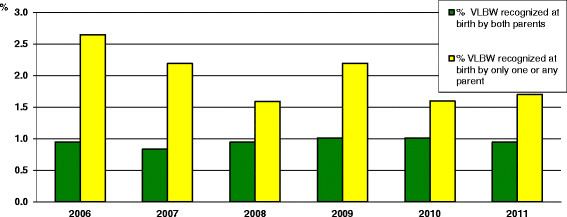
**Rate of very low birth weight infants related to recognition at birth by parents.** Lazio Region of Italy.
